# Current Hospital Topics

**Published:** 1907-06-29

**Authors:** 


### CURRENT HOSPITAL TOPICS.

### A United States and a Canadian General Hospital.

The Roosevelt Hospital, New York.

This hospital provides accommodation for 245 in-patients, and its history of some thirty-six years is instructive. Opened in 1874, an ambulance service was established in 1878; an out-patient department was added in 1881; the gynæcological division was separated from the medical in 1888; and a separate gynæcological theatre was provided in 1890; a private patients' pavilion was erected in 1896, and has proved of immense service to many classes of people in New York; a training school for nurses was organised in 1896; and the accident building, including provision for the treatment of emergency and accident cases, was erected in 1898. In the following year a special ward for sick children with twelve beds, endowed in perpetuity by Miss Bliss, was opened; an office building for the accommodation of the superintendent and his staff was occupied in 1901, when a bureau of information, comprising the records of all kinds connected with the hospital and its work, was established. This brief epitome partially reflects the progressive development of the hospital unit in the United States during the years to which it relates. The Roosevelt Hospital has experienced a grievous loss by the death of Mr. Jas. R. Lathrop, who has been superintendent from 1883; the value of his work is testified to by the Board, who speak of his capacity, devotion to duty, and successful efforts to place the Roosevelt Hospital in the first rank of the hospitals of New York. The report under notice confirms the Board's eulogy of his efficient management, for it differs from many reports in that, instead of the long list of subscribers and donors, each page conveys instructive, detailed, and useful information of a practical character. It is interesting to note, for instance, that as many as 884 patients applying for admission have been rejected in the course of one year, of whom the majority were regarded as unsuitable cases for treatment in an active general hospital. It has been the practice to keep in touch with other medical institutions, and the ambulance service is utilised for the removal of patients to and from the hospital proper, from the patient's home to other hospitals, and from the casualty department to their own homes. Two thousand and thirty-two persons were taken to Bellevue Hospital, the equivalent of our Poor-law infirmaries in New York City. Of 4,404 in-patients 1,348 paid for full or partial board, and 3,056 were treated entirely free. The number of ambulance calls in the year was 5,703, and 111 patients, after being examined at their own homes, were brought to the hospital by the ambulance. Of the 245 beds, 202 were constantly occupied throughout the year. The staff consisted of three house officers, sixteen medical men, and two hundred and thirty-eight attendants of all kinds, including the special nurses. More than three thousand pounds was paid for the services of special nurses by the private patients. The nursing staff, including the superintendents, amounted to seventy-two, of whom twenty-eight were first year, sixteen second year, and nineteen third year probationers. In the out-patient department 16,296 new patients were treated, the estimated cost being £2,000, of which £1,650 was refunded by the patients' payments for prescriptions and dressings, so that the actual cost to the hospital of this department was only about £350. The gross cost of each in-patient per day, calculated on the actual expenses for maintenance of the hospital proper, was roughly nine shillings. Particulars are given of the number of prescriptions dispensed, together with several interesting facts in relation to the cost and working of each department of the hospital.

Montreal General Hospital.

Canada can boast that it possesses the oldest hospital on the American Continent—*i.e.*, the Hôtel Dieu, Quebec, founded in 1636 and burnt in 1755. The Montreal General Hospital has had eighty-five years of useful existence, and its report is issued in the most attractive cover we have seen this year. It consists of greyish-blue cartridge paper, with a five-inch square Latin cross, blocked in sealing-wax red, the arms of which measure one and three quarter inches across. On the cross are printed the name of the hospital, with an impression of its seal, and a summary of the contents of the report. The whole is exceedingly well done and very striking. Like University College Hospital, that in Montreal has now acquired the whole square of land, on a portion of which the hospital was originally built, so that it will thus have a frontage on four streets. It has been wisely determined to remove the present heating, lighting, and laundry departments to a new site at the rear of the hospital. The number of beds is not stated, but the average number occupied was 201, the average residence of each patient twenty-one and one-fifth days, and the average daily cost of an in-patient about six shillings. Of the in-patients, excluding a few foreigners, roughly two-thirds were natives of Canada, and one-third of the British Isles. This is an interesting fact to note in these imperial days. The new out-patients amounted to 14,448. The ambulance was used 1,628 times, the bulk of the cases being brought to the hospital, and 899 admitted as in-patients. The report contains the names of 270 nurses who have graduated from this hospital. The expenditure last year exceeded the income by about £2,200. The hospital has an endowment fund of £20,000, and the reserve fund investments amount to upwards of £70,000 more. London hospital managers might note the fact that the Canadian insurance companies contribute annually to this hospital. It may be noted that nearly as much is spent on pork and pork products as upon mutton, veal, and lamb; that the beef bill is almost as large as that for milk and cream; and that both fresh and canned fish are used in the hospital. The form of the report might be modernised with advantage, as the information it contains is not at present readily available or nearly as interesting as it might be made.

